# Blood–Brain Barrier Disruption in Preclinical Mouse Models of Stroke Can Be an Experimental Artifact Caused by Craniectomy

**DOI:** 10.1523/ENEURO.0343-22.2022

**Published:** 2022-10-24

**Authors:** Che-Wei Liu, Eric Yuhsiang Wang, Hwai-Lee Wang, Kate Hsiurong Liao, Hsiao-Yun Chen, Hank Szuhan Chen, Ted Weita Lai

**Affiliations:** 1 Graduate Institute of Biomedical Sciences, China Medical University, Taichung 404333, Taiwan; 2 School of Medicine, China Medical University, Taichung 404333, Taiwan; 3 Department of Neurosurgery, Taipei Veterans General Hospital, Taipei 112201, Taiwan; 4 Graduate Institute of Clinical Medical Science, China Medical University, Taichung 404333, Taiwan; 5 Department of Anesthesiology, China Medical University Hospital, Taichung 404327, Taiwan; 6 Drug Development Center, China Medical University, Taichung 404333, Taiwan; 7 Translational Medicine Research Center, China Medical University Hospital, Taichung 404327, Taiwan

**Keywords:** blood–brain barrier, cerebral edema, dMCAO, mice, preclinical model, stroke

## Abstract

The pathophysiological features of ischemia-related blood–brain barrier (BBB) disruption are widely studied using preclinical stroke models. However, in many of these models, craniectomy is required to confirm arterial occlusion via laser Doppler flowmetry or to enable direct ligation of the cerebral artery. In the present study, mice were used to construct a distal middle cerebral artery occlusion (dMCAO) model, a preclinical stroke model that requires craniectomy to enable direct ligation of the cerebral artery, or were subjected to craniectomy alone. dMCAO but not craniectomy caused neurodegeneration and cerebral infarction, but both procedures induced an appreciable increase in BBB permeability to Evans blue dye, fluorescein, and endogenous albumin but not to 10 kDa dextran-FITC, leading to cerebral edema. Using rats, we further showed that BBB disruption induced by craniectomy with no evidence of dural tearing was comparable to that induced by craniectomy involving tearing of the dura. In conclusion, our data demonstrated that craniectomy can be a major contributor to BBB disruption and cerebral edema in preclinical stroke models. The implications of this experimental artifact for translational stroke research and preclinical data interpretation are discussed.

## Significance Statement

Craniectomy is often performed in preclinical stroke studies. In the conventional middle cerebral artery occlusion (MCAO) model involving suture insertion, craniectomy is required to allow laser Doppler flowmetry, which is key for ensuring complete occlusion and minimizing suture-induced brain hemorrhage. In various distal MCAO models, craniectomy is required to enable direct ligation of the cerebral arteries. However, in this study, we found that craniectomy is a major cause of BBB disruption in preclinical stroke models. This has major implications for preclinical data interpretation and translational stroke research, as most previous studies (including ours) were conducted under the wrong assumption that BBB disruption and cerebral edema in these models are caused by ischemia/neuronal injuries per se.

## Introduction

Blood–brain barrier (BBB) disruption after an ischemic stroke is widely believed to be a contributing factor to increased stroke severity. To better understand stroke pathogenesis, several groups have researched ischemia-related BBB disruption using different preclinical stroke models; these include the following: (1) the conventional intraluminal suture insertion-induced middle cerebral artery occlusion (MCAO) model, in which a suture is inserted through the external or common carotid artery into the middle cerebral artery and cerebral blood flow is monitored by laser Doppler flowmetry through an open cranial window; and (2) the distal MCAO (dMCAO) model, in which the distal middle cerebral artery is directly occluded via an open cranial window. It is generally assumed that BBB disruption in these animal models reflects the clinical pathophysiology seen in stroke patients.

Recently, it was shown that craniectomy itself, even in the absence of cerebral ischemia induced by MCAO or dMCAO, can cause BBB disruption in a temperature-dependent manner. Specifically, local heating because of drilling can cause BBB disruption in animals undergoing craniectomy (Shoffstall et al., 2018). Moreover, two recent studies showed that BBB disruption caused by dMCAO but not that caused by carbogen inhalation is temperature dependent (Liu et al., 2017; Poon et al., 2020), and that delayed hypothermia completely abolishes BBB disruption induced by dMCAO without affecting ischemic infarction (Liu et al., 2017). These findings raise the intriguing possibility that BBB disruption in preclinical stroke models that require craniectomy can partly be an experimental artifact related to the experimental procedure.

In this study, we compared BBB permeability to Evans blue dye (EBD), fluorescein, 10 kDa dextran-FITC, and endogenous albumin in mice subjected to dMCAO, which requires craniectomy, to that in mice subjected to craniectomy alone without induction of cerebral ischemia. Our data provide evidence that BBB disruption in preclinical stroke models that require craniectomy can, at least in part, be an artifact related to the experimental procedure.

## Materials and Methods

### Animals

Male C57BL/6 mice and Sprague Dawley rats were purchased from the National Laboratory Animal Center (Taipei, Taiwan). They were housed under a 12 h light/dark cycle and provided food and water *ad libitum*. All mice and rats were subjected to experimental procedures at 7–9 weeks of age; the body weight range was 21–34 g for mice and 250–350 g for rats. All experimental procedures were performed in accordance with the ARRIVE guidelines and the Institutional Guidelines for China Medical University (CMU) for the Care and Use of Experimental Animals and were approved by the Institutional Animal Care and Use Committee of CMU (protocol No. 103–224-NH).

### Surgical procedure

Mice and rats were anesthetized by inhalation of isoflurane (1.5% in air) before all surgical procedures. To induce cerebral ischemia, mice were subjected to dMCAO and ipsilateral common carotid arterial occlusion, as described previously (Wang and Lai, 2021). In brief, a small craniectomy was made in the right temporal bone to enable ligation of the distal cortical branch of the middle cerebral artery with a surgical nylon suture, and this procedure was accompanied by reversible ligation of the right common carotid artery using cotton sewing thread. During occlusion, the mice were recovered from anesthesia in a 37°C cage. After 2 h of occlusion, they were reanesthetized and the ligatures around the middle cerebral artery and common carotid artery were removed to achieve complete reperfusion. For mice and rats that underwent craniectomy only, a small craniectomy was made in the right temporal bone at the same location as for dMCAO, and the surgical wound was closed as described above. EBD (4% in saline; 2 ml/kg) was injected into rats via the tail vein to facilitate identification of the dura matter and visualization of dural tearing. Bupivacaine (0.5%) was administered subcutaneously presurgically and postsurgically to relieve surgical pain. A subset of mice was killed 24 h after surgery, and coronal brain sections were stained with 2,3,5-triphenyltetrazolium chloride (TTC) to identify the ischemic brain region or Fluoro-Jade to assess neurodegeneration, as described previously (Liu et al., 2020).

### Quantification of BBB permeability by exogenous tracers

Tracers, including EBD (4% in saline; 2 ml/kg), fluorescein (4% solution in saline; 2 ml/kg), and 10 kDa dextran-FITC (10 mg/ml in PBS; 4 ml/kg), were injected into mice via the tail vein 1 h before tissue collection at 3, 6, 12, 24, 48, or 72 h after dMCAO or craniectomy. At the time of tissue collection, the mice were anesthetized by an overdose of urethane (4 g/kg, i.p.). After blood samples (∼50 μl) were collected, the mice were intracardially perfused with saline to wash out the residual tracer from the circulation, and their brains and livers were collected. The concentrations of tracers in brain, liver, and blood samples were quantified by spectrophotometry, as described previously (Yen et al., 2013).

### Quantification of BBB permeability to endogenous albumin

Mice and rats were killed by an overdose of urethane 24 h after dMCAO or craniectomy and then perfused with cold saline to remove circulating albumin from the blood. Isolated brains were coronally sectioned at 2 mm thickness. Motor cortex tissue from the middle two coronal sections (approximately +2 to +6 mm from λ), where infarct occurred or would have occurred, was isolated for Western blotting as described previously (Liao et al., 2019). In brief, these samples were homogenized in lysis buffer (20 mm Tris, 150 mm NaCl, 1 mm EDTA, and 1% NP-40 adjusted to pH 7.4) containing protease inhibitor (catalog #04693132001, Roche) and centrifuged at 12,000 rpm for 30 min. Proteins (∼30 μg) from the supernatants were heated for 5 min at 100°C, separated by electrophoresis, and transferred to PVDF membranes. After blocking in milk (5% dry milk powder in TBST) for 2 h at room temperature, each membrane was incubated with primary antibodies against α-tubulin (1:5000 in 2% BSA in TBST; catalog #GTX628802, Genetex) and albumin (1:5000 in 2% BSA in TBST; catalog #ab106582, Abcam) overnight at 4°C, washed with TBST, and incubated with goat anti-mouse IgG (1:1000 in 2% BSA in TBST; catalog #GTX213111-01, Genetex) and goat anti-chicken IgY (1:1000 in 2% BSA in TBST; catalog #ab97135, Abcam) secondary antibodies. Western blot images were taken using a ChemiDoc XRS+ System (BIO-RAD).

### Quantification of cerebral edema

Mice were killed by an overdose of urethane 24 h after dMCAO or craniectomy, and isolated brains were weighed before and after drying in a 60°C oven overnight. The water (edema) content was calculated by subtracting the dry weight of each hemisphere from its wet weight.

### Statistical analysis

The data are presented as the mean ± SEM. Tracer and albumin extravasation into the brain and cerebral edema after dMCAO or craniectomy was compared by mixed-effects analysis or two-way repeated-measures ANOVA (matching brain regions from the same animal) followed by Holm–Sidak multiple-comparisons test. Tracer extravasation into the liver and the concentration of tracer remaining in the blood at various time points after dMCAO were compared by one-way ANOVA followed by Holm–Sidak multiple-comparisons test. Tracer extravasation into the liver and the concentration of tracer remaining in the blood after craniectomy were compared by unpaired *t* test.

## Results

### dMCAO and craniectomy increase BBB permeability to EBD

**Figure 1.**
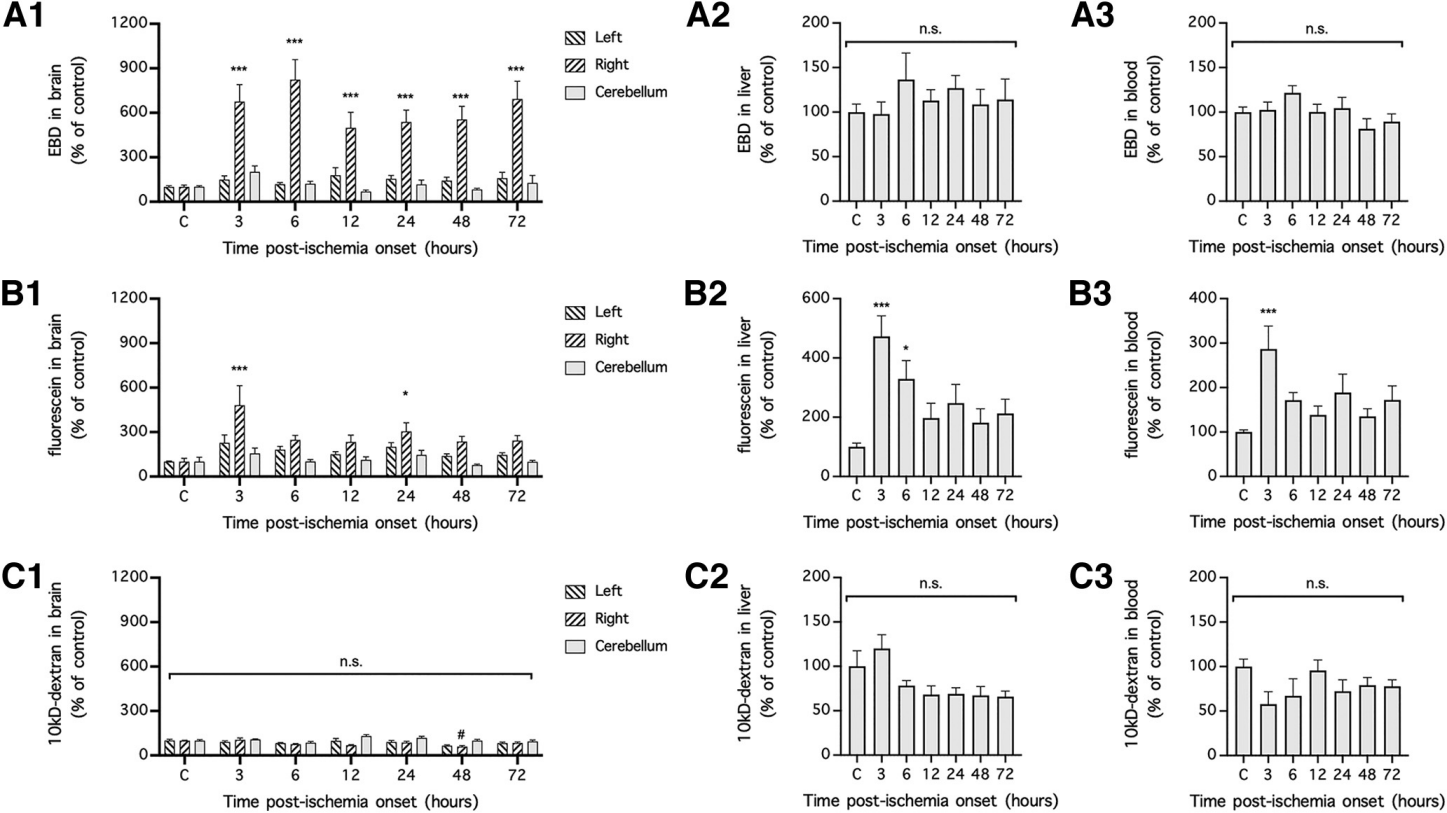
dMCAO unilaterally increased BBB permeability to EBD and fluorescein in mice. ***A1–C3***, Focal cerebral ischemia of the right motor cortex was induced in mice, and the mice were intravenously injected with EBD (***A1–A3***), fluorescein (***B1–B3***), or 10 kDa dextran-FITC (***C1–C3***) at the indicated time points (C, control). Extravasation of the circulating tracers into the left (contralateral, nonischemic) hemisphere, right (ipsilateral, ischemic) hemisphere, and cerebellum was used as a measure of BBB permeability, and extravasation of the tracers into the liver and the concentrations of the tracers remaining in the blood were used as measures of tracer stability in the circulation. In ***A1–A3***, *n* = 10 mice/time point except for the cerebellum, for which *n* = 5 mice/time point. In ***B1–B3***, *n* = 5–7 mice/time point. In ***C1–C3***, *n* = 5 mice/time point. The concentrations of tracers in the brain were compared by mixed-effects ANOVA (***A1***) or two-way repeated-measures ANOVA (matching brain regions from the same mouse; ***B1*** and ***C1***) followed by Holm–Sidak multiple-comparisons test. The concentrations of tracers in the liver (***A2***, ***B2***, and ***C2***) and blood (***A3***, ***B3***, and ***C3***) were compared with one-way ANOVA followed by Holm–Sidak multiple-comparisons test. *p <* *0.05, ***p <* *0.001, compared with the control. n.s., No significant difference.

To compare BBB disruption induced by dMCAO and that resulting from craniectomy, we first identified the suitable intravenous tracers and the time frame for quantifying BBB disruption in mice subjected to dMCAO. When EBD (4% in saline; 2 ml/kg) was intravenously injected and allowed to circulate for 1 h in the blood, the tracer became concentrated in the ipsilateral (right) hemispheres and to a lesser extent in the contralateral (left) hemispheres and in cerebella of the mice 3, 6, 12, 24, 48, and 72 h after dMCAO (*n* = 10 mice/time point except for cerebellum, for which *n* = 5 mice/time point; *p *=* *0.0201 for differences in tracer extravasation over time, *p *<* *0.0001 for differences between brain regions, mixed-effects analysis; *p *<* *0.001 compared with the control for all time points, Holm–Sidak multiple-comparisons test; Fig. 1*A1*). Unlike the extravasation of EBD into the ischemic brain region, there was no change in EBD concentrations in the liver and blood following dMCAO (*n* = 10 mice/time point; *p *=* *0.7368 for the liver, *p *=* *0.1005 for the blood, one-way ANOVA; *p *>* *0.05 compared with the control for all time points for the liver and blood, Holm–Sidak multiple-comparisons test; Fig. 1*A2*,*A3*). Similarly, when fluorescein (4% in saline; 2 ml/kg) was intravenously injected and allowed to circulate for 1 h in the blood, the tracer became concentrated in the ipsilateral (right) hemispheres and to a lesser extent in the contralateral (left) hemispheres and cerebella of the mice 3 and 24 h after induction of dMCAO (*n* = 5–7 mice/time point; *p *=* *0.0160 for differences in tracer extravasation over time, *p *<* *0.0001 for differences between brain regions, two-way repeated-measures ANOVA of data from matching brain regions from the same mouse; *p *<* *0.0001 compared with the control for the 3 h time point, *p *=* *0.0106 compared with the control for the 24 h time point, Holm–Sidak multiple-comparisons test; Fig. 1*B1*). However, given that fluorescein concentrations in the liver and blood were also significantly increased at 3 h after ischemia onset (*n* = 5–7 mice/time point; *p *=* *0.0008 for the liver, *p *=* *0.0066 for the blood, one-way ANOVA; *p *= 0.0001 compared with the control for the liver, *p *=* *0.0009 compared with the control for the blood, Holm–Sidak multiple-comparisons test; Fig. 1*B2*,*B3*), fluorescein extravasation into the brain at this time point was likely exacerbated by the increased concentration of fluorescein in the blood. Unlike for EBD and fluorescein, when 10 kDa dextran-FITC (4% in saline; 2 ml/kg) was intravenously injected and allowed to circulate for 1 h in blood, there was no increase in its concentration in the ipsilateral (right) hemispheres of mice subjected to dMCAO and those of mice not subjected to dMCAO (*n* = 5 mice/time point; *p *>* *0.05 compared with the control for the ipsilateral hemisphere at 3, 6, 12, 24, 72 h, Holm–Sidak multiple-comparisons test) and a marginal decrease in tracer concentration at the 48 h time point (*n* = 5 mice/time point; *p *=* *0.0036 for differences in tracer extravasation over time, *p *<* *0.0001 for differences between brain regions, two-way repeated-measures ANOVA of data from matching brain regions from the same mouse; *p *=* *0.0194 compared with the control, Holm–Sidak multiple-comparisons test; Fig. 1*C1*). There was little or no change in 10 kDa dextran-FITC concentrations between liver and blood samples from mice subjected to dMCAO and those from mice not subjected to dMCAO (*n* = 5 mice/time point; *p *=* *0.0095 for the liver, *p *=* *0.2197 for the blood, one-way ANOVA; *p *>* *0.05 compared with the control for all time points for the liver and blood, Holm–Sidak multiple-comparisons test; Fig. 1*C2*,*C3*). Based on the above experiments, we concluded that EBD and fluorescein, but not 10 kDa dextran-FITC, are suitable for assessing BBB disruption in mice 24 h after dMCAO.

**Figure 2.**
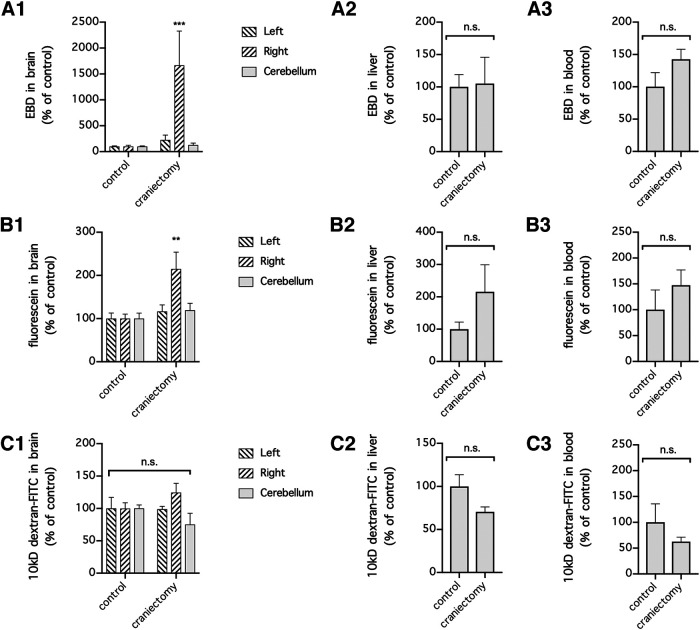
Craniectomy in mice unilaterally increased BBB permeability to EBD and fluorescein. ***A1–C3***, Mice were subjected to craniectomy above the right motor cortex and intravenously injected with EBD (***A1–A3***), fluorescein (***B1–B3***), or 10 kDa dextran-FITC (***C1–C3***) 24 h later. Extravasation of the circulating tracers into the left (contralateral) hemisphere, right (ipsilateral) hemisphere, and cerebellum was used as a measure of BBB permeability, and extravasation of the tracers into the liver and the concentrations of the tracers remaining in the blood were used as measures of tracer stability in the circulation. *n* = 8 mice/group for EBD (***A1–A3***) and *n* = 5 mice/time point for fluorescein (***B1–B3***) and 10 kDa dextran-FITC (***C1–C3***). The concentrations of tracers in the brain were compared by two-way repeated-measures ANOVA (matching brain regions from the same mouse) followed by Holm–Sidak multiple-comparisons test (***A1***, ***B1***, and ***C1***). The concentrations of tracers in the liver (***A2***, ***B2***, and ***C2***) and blood (***A3***, ***B3***, and ***C3***) were compared with unpaired *t* test. **p <* *0.01, ***p <* *0.001, compared with the control. n.s., No significant difference.

To examine whether craniectomy can similarly cause cerebral extravasation of EBD and fluorescein 24 h after surgery, we subjected mice to craniectomy. The mice were subjected to a procedure similar to dMCAO induction but without ligation of the middle cerebral artery. Interestingly, similar to after dMCAO, after EBD and fluorescein were injected and allowed to circulate in the blood for 1 h, they became concentrated in the ipsilateral (right) hemispheres and to a lesser extent in the contralateral (left) hemispheres and cerebella of the mice 24 h after craniectomy (*n* = 8 mice/group for EBD; *n* = 5 mice/group for fluorescein; *p *=* *0.0425 for differences in EBD extravasation over time, *p *=* *0.0068 for differences in EBD extravasation between brain regions, *p *=* *0.0499 for differences in fluorescein extravasation over time, *p *=* *0.0105 for differences in fluorescein extravasation between brain regions, two-way repeated-measures ANOVA of data from matching brain regions from the same mouse; *p *=* *0.0006 compared with the control for EBD in the ipsilateral hemisphere, *p *=* *0.9834 compared with the control for EBD in the contralateral hemisphere, *p *=* *0.9998 compared with the control for EBD in the cerebellum, *p *=* *0.0015 compared with the control for fluorescein in the ipsilateral hemisphere, *p *=* *0.9195 compared with the control for fluorescein in the contralateral hemisphere, *p *=* *0.8788 compared with the control for fluorescein in the cerebellum, Holm–Sidak multiple-comparisons test; Fig. 2*A1*,*B1*). There was no significant change in the concentrations of EBD or fluorescein in the liver and blood (*p *=* *0.9127 for EBD in the liver, *p *=* *0.1347 for EBD in the blood, *p *=* *0.2194 for fluorescein in the liver, *p *= 0.3560 for fluorescein in the blood, unpaired *t* test; Fig. 2*A2*,*A3*,*B2*,*B3*). In contrast to cerebral extravasation of EBD and fluorescein and similar to what we found in mice subjected to dMCAO, there was no significant difference in the concentrations of 10 kDa dextran in the brain, liver, and blood in mice subjected to craniectomy (*n* = 5 mice/group; *p *=* *0.9739 for differences in tracer extravasation over time, *p *=* *0.0681 for differences between brain regions, two-way repeated-measures ANOVA of data from matching brain regions from the same mouse; *p *=* *0.4271 compared with the control for the ipsilateral hemisphere, *p *=* *0.9532 compared with the control for the contralateral hemisphere, *p *= 0.4271 compared with the control for the cerebellum, Holm–Sidak multiple-comparisons test; *p *=* *0.0803 for the liver, *p *=* *0.3401 for the blood, unpaired *t* test; Fig. 2*C1–C3*). Together, our data showed that craniectomy alone causes unilateral cerebral extravasation of circulating EBD and fluorescein into the ipsilateral hemisphere and can therefore contribute to the BBB disruption in preclinical mouse models of stroke involving craniectomy.

### Craniectomy causes BBB disruption without neuronal injury

**Figure 3.**
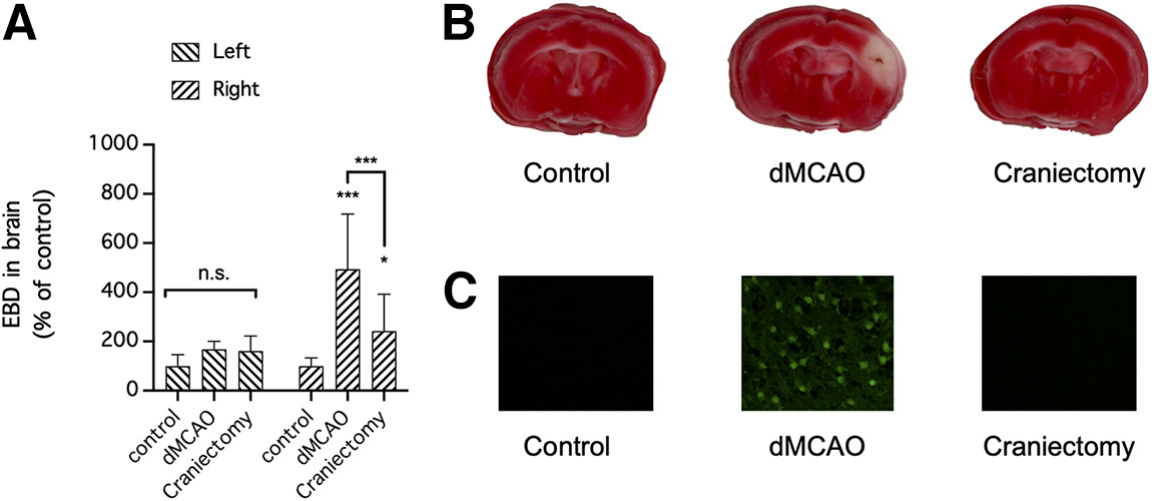
Craniectomy contributed to BBB disruption but not to cerebral infarction and neurodegeneration in a preclinical mouse model of stroke. ***A***, Mice were subjected to focal cerebral ischemia induced by dMCAO or craniectomy above the right motor cortex and intravenously injected with EBD 24 h later. Extravasation of the circulating tracers into the left (contralateral, nonischemic) hemisphere and right (ipsilateral, ischemic) hemisphere was used as a measure of BBB permeability (*n* = 10 mice/group). The concentrations of tracers in the brain were compared by two-way repeated-measures ANOVA (matching brain regions from the same mouse) followed by Holm–Sidak multiple-comparisons test. *p <* *0.05; ***p <* *0.001, compared with the control or for dMCAO versus craniectomy. n.s., No significant difference. ***B***, ***C***, Mice were subjected to focal cerebral ischemia induced by dMCAO or craniectomy above the right motor cortex and killed 24 h later to prepare coronal brain sections, which were stained with TTC or Fluoro-Jade to identify infarcted brain regions (***B***) and assess neurodegeneration (***C***), respectively. Each image in ***B*** and ***C*** is representative of *n* = 3 mice/group; there were no noticeable differences between individual mice from the same group.

To determine whether craniectomy is the sole cause of BBB disruption in mice subjected to dMCAO, mice were subjected to either dMCAO involving craniectomy or craniectomy alone and injected with EBD 24 h after surgery (Fig. 3*A*). After EBD was allowed to circulate in the blood for 1 h, the concentration of EBD was increased in the ipsilateral (right) hemisphere and to a lesser extent in the contralateral (left) hemisphere in mice subjected to either surgical procedure compared with control mice that did not undergo surgery (*n* = 10 mice/group; *p *<* *0.0001 for differences in tracer extravasation over time, *p *<* *0.0001 for differences between brain regions, two-way repeated-measures ANOVA of data from matching brain regions from the same mouse; ipsilateral hemisphere: *p *<* *0.0001 compared with the control for dMCAO, *p *=* *0.0082 compared with the control for craniectomy; contralateral hemisphere: *p *=* *0.4835 compared with the control for both dMCAO and craniectomy; Holm–Sidak multiple-comparisons test). Nevertheless, EBD extravasation into the ipsilateral (right) hemisphere in mice subjected to dMCAO was greater than that in mice subjected to craniectomy alone (*p *<* *0.0001 for dMCAO vs craniectomy, Holm–Sidak multiple-comparisons test; Fig. 3*A*). Given that our data showed that craniectomy contributed to dMCAO-mediated BBB disruption, we examined whether craniectomy also contributes to dMCAO-induced brain infarction and neuronal injury (Fig. 3*B*,*C*). Mice were subjected to dMCAO or craniectomy alone, and 24 h after surgery their brains were collected and stained with TTC, which stains healthy brain tissues red, to visualize the infarcted area and Fluoro-Jade, which selectively stains degenerating neurons. Although both surgical procedures caused BBB disruption, dMCAO but not craniectomy alone caused marked cerebral infarction (Fig. 3*B*) and neurodegeneration (Fig. 3*C*). Therefore, craniectomy can affect BBB permeability without causing infarction or neurodegeneration.

### dMCAO and craniectomy increase BBB permeability to endogenous albumin

**Figure 4.**
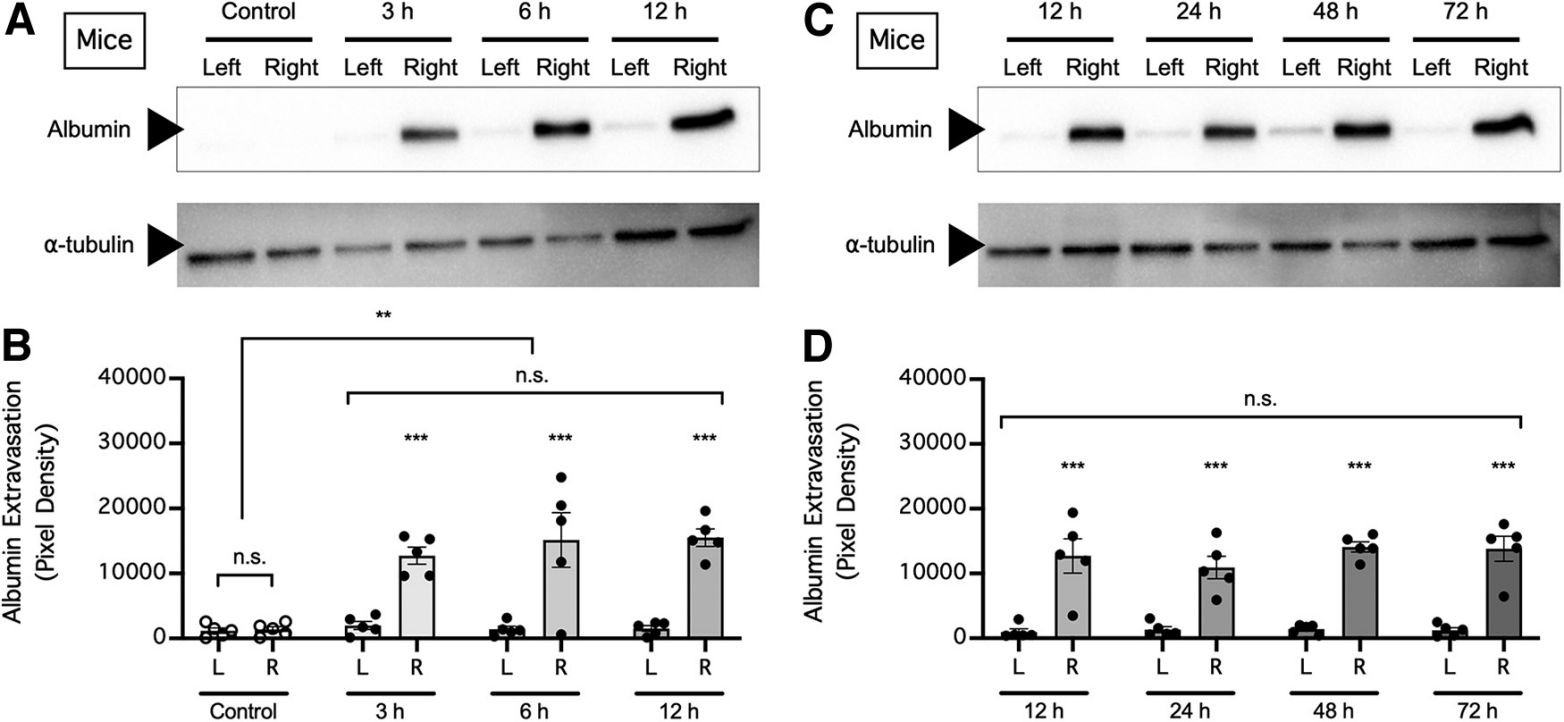
dMCAO unilaterally increased cerebral extravasation of endogenous albumin in mice. ***A***, Mice were subjected to focal cerebral ischemia of the right motor cortex and perfused to remove circulating albumin from the blood 3, 6, or 12 h later, and extravasation of albumin into the left (contralateral, nonischemic) and right (ipsilateral, ischemic) hemispheres was assessed by Western blotting. Control mice exhibited no cerebral ischemia. ***B***, Summarized results for ***A***. ***C***, Mice were subjected to focal cerebral ischemia of the right motor cortex and perfused to remove circulating albumin from the blood 12, 24, 48, or 72 h later, and extravasation of albumin into the left (contralateral, nonischemic) and right (ipsilateral, ischemic) hemispheres was evaluated by Western blotting. ***D***, Summarized result for ***C***. In ***B*** and ***D***, albumin extravasation was compared by two-way repeated-measures ANOVA (matching brain regions from the same mouse) followed by Holm–Sidak multiple-comparisons test. **p <* *0.01, ***p <* *0.001. n.s., No significant difference.

**Figure 5.**
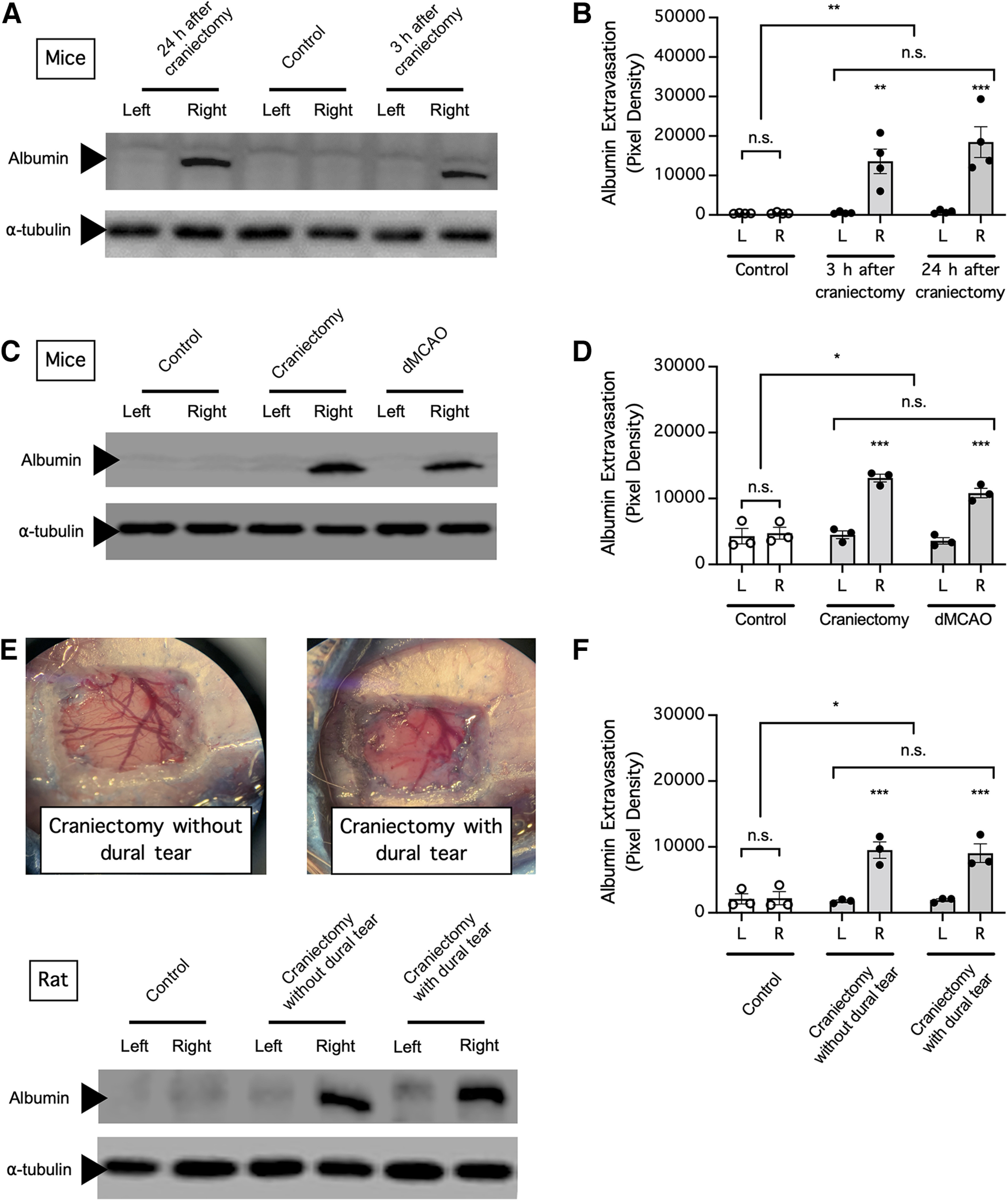
Craniectomy contributed to increased cerebral extravasation of endogenous albumin in a preclinical stroke model independent of dural tearing. ***A***, Mice were subjected to craniectomy above the right motor cortex and perfused to remove circulating albumin from the blood 3 or 24 h later, and extravasation of albumin into the left (contralateral) and right (ipsilateral) hemispheres was assessed by Western blotting. ***B***, Summarized result for ***A***. ***C***, Mice were subjected to focal cerebral ischemia induced by dMCAO to or craniectomy above the right motor cortex and perfused to remove circulating albumin from the blood 24 h later, and extravasation of albumin into the left (contralateral, nonischemic) and right (ipsilateral, ischemic) hemispheres was assessed by Western blotting. ***D***, Summarized data for ***C***. ***E***, To determine the effect of dural tearing, rats were injected with Evans blue dye to facilitate identification of the dura mater during craniectomy and were perfused to remove circulating albumin from the blood 24 h later; and extravasation of albumin into the left (contralateral, nonischemic) and right (ipsilateral, ischemic) hemispheres was evaluated by Western blotting. ***F***, Summarized data for ***E***. In ***B***, ***D***, and ***F***, albumin extravasation was compared by two-way repeated-measures ANOVA (matching brain regions from the same mouse) followed by Holm–Sidak multiple-comparisons test. *p <* *0.05, **p <* *0.01, ***p <* *0.001. n.s., No significant difference.

Taking into consideration the various limitations of using exogenous tracers, such as EBD and fluorescein, in studying BBB permeability, we asked whether dMCAO and craniectomy also increase cerebral extravasation of endogenous circulating albumin in mice. In mice subjected to dMCAO, extravasation of endogenous albumin into the ipsilateral (right) hemisphere was observed in the early phase (*n* = 5 mice/time point; *p *=* *0.0033 for differences in albumin extravasation over time, *p *<* *0.0001 for differences between brain regions, two-way repeated-measures ANOVA of data from matching brain regions from the same mouse; *p *>* *0.9999 for the ipsilateral hemisphere compared with the contralateral hemisphere in the control group, *p *=* *0.0004 for the ipsilateral hemisphere compared with the contralateral hemisphere at 3 h post-ischemia onset, *p *<* *0.0001 for the ipsilateral hemisphere compared with the contralateral hemisphere at 6 h post-ischemia onset, *p *<* *0.0001 for the ipsilateral hemisphere compared with the contralateral hemisphere at 12 h post-ischemia onset, Holm–Sidak multiple-comparisons test; Fig. 4*A*,*B*) and the later phase (12, 24, 48, and 72 h) of ischemia (*n* = 5 mice/time point; *p *=* *0.7238 for differences in albumin extravasation over time, *p *<* *0.0001 for differences between brain regions, two-way repeated-measures ANOVA of data from matching brain regions from the same mouse; *p *<* *0.0001 for the ipsilateral hemisphere compared with the contralateral hemisphere at 12 h post-ischemia onset, *p *=* *0.0001 for the ipsilateral hemisphere compared with the contralateral hemisphere at 24 h post-ischemia onset, *p *<* *0.0001 for the ipsilateral hemisphere compared with the contralateral hemisphere at 48 h post-ischemia onset, *p *<* *0.0001 for the ipsilateral hemisphere compared with the contralateral hemisphere at 72 h post-ischemia onset, Holm–Sidak multiple-comparisons test; Fig. 4*C*,*D*); in comparison, little endogenous albumin was found in brain samples from control mice or in the contralateral (left) hemispheres of mice subjected to dMCAO. Likewise, craniectomy without cerebral ischemia caused profound extravasation of endogenous albumin into the ipsilateral (right) but not the contralateral (left) hemisphere in mice (*n* = 4 mice/time point; *p *=* *0.0053 for differences in albumin extravasation over time, *p *=* *0.0001 for differences between brain regions, two-way repeated-measures ANOVA of data from matching brain regions from the same mouse; *p *= 0.9805 for the ipsilateral hemisphere compared with the contralateral hemisphere in the control group, *p *=* *0.0021 for the ipsilateral hemisphere compared with the contralateral hemisphere at 3 h postcraniectomy, *p *=* *0.0004 for the ipsilateral hemisphere compared with the contralateral hemisphere at 24 h postcraniectomy, Holm–Sidak multiple-comparisons test; Fig. 5*A*,*B*). Interestingly, we found no significant difference between albumin extravasation by craniectomy compared with that by dMCAO (*n* = 3 mice/group; *p *=* *0.0197 for differences in albumin extravasation between control and treatment groups, *p *<* *0.0001 for differences between brain regions, two-way repeated-measures ANOVA of data from matching brain regions from the same mouse; *p *=* *0.4851 for the ipsilateral hemisphere compared with the contralateral hemisphere in the control group, *p *<* *0.0001 for the ipsilateral hemisphere compared with the contralateral hemisphere in the craniectomy group, *p *<* *0.0001 for the ipsilateral hemisphere compared with the contralateral hemisphere in the dMCAO group, *p *=* *0.4651 for the craniectomy group compared with the dMCAO group, Holm–Sidak multiple-comparisons test; Fig. 5*C*,*D*). To determine whether BBB disruption caused by craniectomy was because of accidental injury to the dura mater underneath the cranium, we injected rats with EBD so that the dura could be easily identified after craniectomy and subjected these rats to craniectomy with or without intentional dural tearing (Fig. 5*E*). We found that both craniectomy with dural tearing and craniectomy without dural tearing resulted in profound extravasation of endogenous albumin into the ipsilateral (right) but not the contralateral (left) hemisphere in rats (*n* = 3 rats/group; *p *=* *0.0287 for differences in albumin extravasation between treatment groups, *p *=* *0.0002 for differences between brain regions, two-way repeated-measures ANOVA of data from matching brain regions from the same mouse; *p *=* *0.9996 for the ipsilateral hemisphere compared with the contralateral hemisphere in the control group, *p *=* *0.0012 for the ipsilateral hemisphere compared with the contralateral hemisphere in the craniectomy without dural tearing group, *p *=* *0.0019 for the ipsilateral hemisphere compared with the contralateral hemisphere in the craniectomy with dural tearing group, Holm–Sidak multiple-comparisons test; Fig. 5*E*,*F*). Together, these data supported the notion that craniectomy alone can disrupt the ability of the BBB to prevent circulating plasma proteins from entering the brain and that dural tearing is not responsible for BBB disruption following craniectomy.

**Figure 6.**
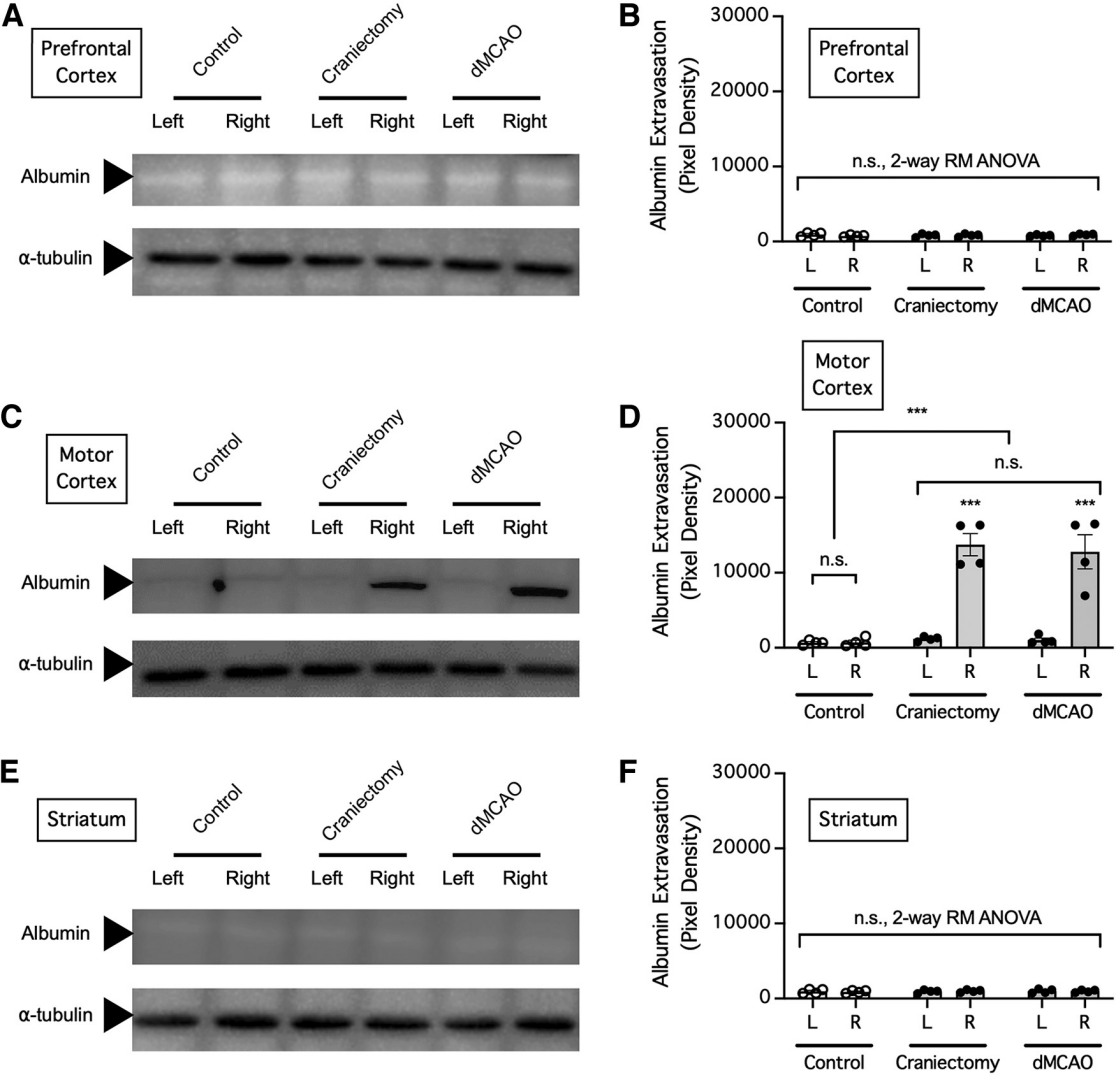
BBB disruption caused by craniectomy was region specific. ***A–F***, Mice were subjected to craniectomy above the right motor cortex and perfused to remove circulating albumin from the blood 24 h later, and extravasation of albumin into the left (contralateral) and right (ipsilateral) prefrontal cortex (***A***, ***B***), motor cortex (***C***, ***D***), and striatum (***E***, ***F***) was assessed by Western blotting. ***B***, Summarized result for ***A***. ***D***, Summarized result for ***C***. ***F***, Summarized result for ***E***. In ***B***, ***D***, and ***F***, albumin extravasation was compared by two-way repeated-measures ANOVA (matching brain regions from the same mouse) followed by Holm–Sidak multiple-comparisons test. ***p <* *0.001. n.s., no significant difference.

To determine the regional specificity of BBB disruption caused by craniectomy, mice were subjected to either dMCAO to or craniectomy above the right motor cortex and extravasation of endogenous albumin into the ipsilateral and contralateral prefrontal cortex, motor cortex, and striatum was determined (Fig. 6*A–F*). Consistent with the above findings, craniectomy without cerebral ischemia caused profound extravasation of endogenous albumin into the ipsilateral (right) but not the contralateral (left) motor cortex, and we found no significant difference between albumin extravasation by craniectomy compared with that by dMCAO (*n* = 4 mice/group; *p *=* *0.0005 for differences in albumin extravasation between control and treatment groups, *p *<* *0.0001 for differences between brain regions, two-way repeated-measures ANOVA of data from matching brain regions from the same mouse; *p *>* *0.9999 for the ipsilateral motor cortex compared with the contralateral motor cortex in the control group, *p *<* *0.0001 for the ipsilateral motor cortex compared with the contralateral motor cortex in the craniectomy group, *p *<* *0.0001 for the ipsilateral motor cortex compared with the contralateral motor cortex in the dMCAO group, *p *=* *0.6525 for the craniectomy group compared with the dMCAO group, Holm–Sidak multiple-comparisons test; Fig. 6*C*,*D*). However, there was no extravasation of endogenous albumin into the ipsilateral (right) or the contralateral (left) prefrontal cortex (Fig. 6*A*,*B*) or striatum (Fig. 6*E*,*F*; *n* = 4 mice/group; *p *=* *0.9581, 0.9159 for differences in albumin extravasation between control and treatment groups for prefrontal cortex and striatum, respectively, *p *=* *0.2481, 0.9887 for differences between left and right brain regions for prefrontal cortex and striatum, respectively, two-way repeated-measures ANOVA of data from matching brain regions from the same mouse). Therefore, craniectomy did not cause noticeable BBB disruption in more distant cortical surfaces, such as that in the prefrontal region, or in deeper brain structures like the striatum.

### dMCAO and craniectomy cause cerebral edema

**Figure 7.**
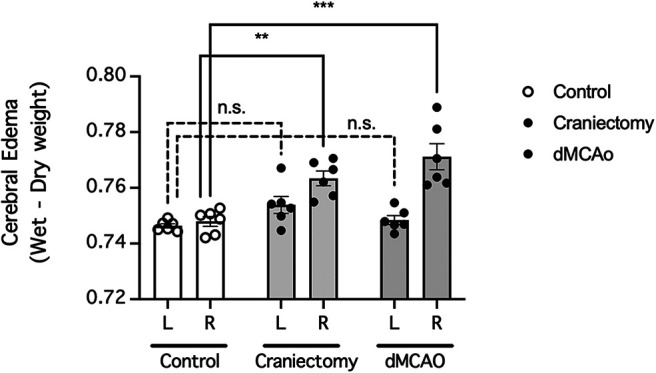
Craniectomy contributed to cerebral edema in a preclinical mouse model of stroke. Mice were subjected to focal cerebral ischemia induced by dMCAO or craniectomy above the right motor cortex and water (edema) contents of their brains were determined 24 h later. *n* = 6 mice/group. Cerebral edemas were compared by two-way repeated-measures ANOVA (matching brain regions from the same mouse) followed by Holm–Sidak multiple-comparisons test. **p <* *0.01, ***p <* *0.001, compared with the control group. n.s., No significant difference.

Given the prominent increase in extravasation of endogenous albumin (Fig. 5*A*,*B*), which increases osmotic pressure, we asked whether dMCAO and craniectomy also cause cerebral edema in mice. Indeed, craniectomy without cerebral ischemia caused significant ipsilateral (right) hemispheric edema but not contralateral (left) hemispheric edema in mice (*n* = 6 mice/group; *p *=* *0.0002 for differences in cerebral edema between treatment groups, *p *=* *0.0003 for differences in cerebral edema between brain regions, two-way repeated-measures ANOVA of data from matching brain regions from the same mouse; *p *=* *0.1720 for craniectomy compared with control for the contralateral hemisphere, *p *=* *0.0011 for craniectomy compared with control for the ipsilateral hemisphere, Holm–Sidak multiple-comparisons test; Fig. 7). Similarly, dMCAO caused significant ipsilateral (right) hemispheric edema but not contralateral (left) hemispheric edema in mice (*n* = 6 mice/group; *p *=* *0.9349 for craniectomy compared with control for the contralateral hemisphere, *p *<* *0.0001 for craniectomy compared with control for the ipsilateral hemisphere, Holm–Sidak multiple-comparisons test; Fig. 7). Therefore, our data showed that craniectomy alone causes cerebral edema and can therefore contribute to the cerebral edema observed in preclinical mouse models of stroke involving craniectomy.

## Discussion

BBB disruption is widely believed to contribute to ischemic brain injury after stroke (Olsson et al., 1971; Albayrak et al., 1997; Shi et al., 2016b). Consistent with this belief, the severity of BBB disruption has been shown to correlate with the extent of ischemic brain injury after stroke (Olsson et al., 1971; Shi et al., 2016a; Haley and Lawrence, 2017; Liu et al., 2017), and drugs that seemingly prevent ischemic BBB disruption also protect the brain against neuronal injury in preclinical stroke models (Liu et al., 2000; Zhou et al., 2014). Therefore, to better understand stroke pathogenesis, several research laboratories have focused on identifying circulating proteins and small molecules that extravasate more readily into the ischemic hemisphere than the nonischemic hemisphere in preclinical stroke models. In this study, among the exogenous tracers injected into the circulation, we found that EBD, which binds to albumin to mimic a protein tracer, was extravasated most readily into the ischemic hemisphere than the nonischemic hemisphere in mice subjected to dMCAO; in comparison, fluorescein less readily extravasated into the ischemic brain, and 10 kDa dextran-FITC did not appear to extravasate into the brain after cerebral ischemia. Similar to EBD, endogenous albumin more readily extravasated into the ischemic hemisphere than the nonischemic hemisphere in mice subjected to dMCAO. Our findings are consistent with those of several previous studies showing that EBD and albumin extravasation is substantially increased but that the extravasation of Ig or small molecules is only moderately increased or not increased in the early phases in preclinical stroke models (Olsson et al., 1971; Belayev et al., 1996; Aoki et al., 2002; Fernández-López et al., 2012; Jackman et al., 2013; Knowland et al., 2014; Liu et al., 2017). Surprisingly, however, we found that craniectomy alone also substantially increased the extravasation of EBD and endogenous albumin but only moderately increased the extravasation of fluorescein into the ipsilateral hemisphere compared with the contralateral hemisphere; similar to the situation after dMCAO, 10 kDa dextran-FITC did not appear to extravasate into the brain after craniectomy. Therefore, our findings demonstrated that craniectomy can partly contribute to BBB disruption in preclinical stroke models constructed by a procedure that involves craniectomy.

The translational failure of experimental stroke treatments in clinical trials has often been attributed to the inability of preclinical animal models to properly reflect stroke pathogenesis in patients. For example, although the majority of stroke patients are hyperglycemic (Scott et al., 1999), preclinical stroke studies are usually performed using only normoglycemic animals. Surprisingly, we recently found that rather than providing neuroprotection, several experimental stroke treatments exacerbate stroke injury in hyperglycemic mice (Wang and Lai, 2021). Likewise, although the majority of stroke trial participants are admitted to the hospital during the daytime (human circadian wake cycle), one recent study reported that several experimental stroke treatments only exert neuroprotective effects in rodent stroke models when performed during the circadian sleep phase, but not during the circadian wake phase (Esposito et al., 2020). Furthermore, although human stroke patients do not typically experience hypothermia whether or not they take antiexcitotoxic agents, antiexcitotoxic agents have been shown to be particularly neuroprotective under hypothermic conditions in experimental animals (Gill et al., 1987; Ikonomidou et al., 1989; Buchan and Pulsinelli, 1990; Alkan et al., 2001) but less neuroprotective or not neuroprotective under normothermic or spontaneous hyperthermic conditions in which antiexcitotoxic agents do not influence body temperature (Buchan and Pulsinelli, 1990; Corbett et al., 1990; Warner et al., 1991; Memezawa et al., 1995; Gerriets et al., 2003; Liu et al., 2020, 2021). Our present finding that BBB disruption in preclinical stroke models can be partly an experimental artifact caused by craniectomy may be another reason for the translational failure of experimental stroke treatments. For example, the contribution of craniectomy to the degree of BBB disruption could have a crucial effect on the delivery, and hence the therapeutic efficacy, of experimental treatments in preclinical stroke studies. This could especially have an impact on neuroprotective peptides that may not be readily BBB permeable, such as nerinetide, a therapeutic peptide that recently performed poorly in a phase III stroke trial despite promising results in preclinical stroke studies (Hill et al., 2020). Additionally, artificial exacerbation of BBB disruption induced by craniectomy in preclinical models can augment the apparent therapeutic effect of experimental treatments that act by minimizing injuries caused by BBB disruption, leading investigators to misinterpret their actual benefits to stroke patients in the clinic.

Of the many exogenous tracers used for measuring BBB permeability, EBD is the most commonly used for evaluating BBB disruption in preclinical stroke models (Olsson et al., 1971; Belayev et al., 1996; Aoki et al., 2002; Fernández-López et al., 2012; Jackman et al., 2013; Knowland et al., 2014; Liu et al., 2017) and has been shown to be more suitable than other tracers for this purpose (Knowland et al., 2014). In this study, EBD also appeared to be more reliable than other exogenous tracers for detecting BBB disruption after stroke. Unlike EBD, fluorescein and dextran were previously reported to be quickly removed from the blood by the kidney, and drugs or interventions that interfere with renal clearance can cause systemic accumulation of these tracers in the blood, brain, and peripheral organs, leading investigators to misinterpret the effect of these treatments on BBB permeability to these tracers (Cheng et al., 2016). Unfortunately, in many preclinical stroke studies that use exogenous tracers to measure BBB permeability, the concentrations of the tracers in the blood and peripheral organs are often not measured. In the present study, we found that the fluorescein concentration in the brain was increased at 3 h postischemia, but this was accompanied by an increase in fluorescein concentrations in the blood and liver. Therefore, the increase in the fluorescein concentration in the brain at this time point does not necessarily reflect a change in BBB permeability. Our findings emphasize the importance of measuring tracer concentrations in the blood and peripheral organs when using exogenous tracers to evaluate BBB permeability. Finally, we showed that, in addition to exogenous tracers, endogenous albumin, to which EBD normally binds, is a circulating protein that is extravasated into the ipsilateral ischemic hemisphere but not the nonischemic hemisphere in our preclinical stroke model. The use of endogenous albumin to measure BBB permeability may alleviate the concerns of some investigators regarding the use of exogenous BBB tracers, including EBD, in preclinical stroke studies. Interestingly, a recent *in vitro* assay showed that EBD-bound albumin is much more permeable across a monolayer of brain endothelial cells compared with unbound albumin (Wu et al., 2021). Therefore, although EBD is commonly used as an albumin tracer, the BBB permeability of EBD injected into blood *in vivo* can only reflect the permeability of EBD-bound albumin and not that of native albumin in non-EBD-treated animals.

The discovery reported in this study was largely serendipitous as we initiated the study without anticipating that craniectomy alone would cause such profound BBB disruption, and the lack of proper control for some experimental variables likely accounted for some of the data variabilities. For example, there was considerable variability in how much BBB disruption was detected in the mouse brain after craniectomy. For instance, craniectomy increased EBD in the brain by ∼1500% in Figure 2*A*, but only increased EBD in the brain by ∼200% in Figure 3*A*. One possible explanation for this difference is that these experiments were performed by different investigators who were used to different drilling speeds, which can cause different drilling temperatures and different degrees of BBB disruption (Shoffstall et al., 2018), when doing the craniectomy. Unfortunately, given the serendipitous nature of this study, in which the craniectomy group was initially included only as part of a control experiment, we did not account for differences in drilling speed in the experimental design. In addition, although the increase in EBD extravasation was more severe after dMCAO than after craniectomy alone, there was no difference in how much albumin extravasation was increased after the two procedures. When considering the fact that EBD-bound albumin and unbound albumin behave very differently across a monolayer of brain endothelial cells (Wu et al., 2021), one possible explanation for this difference is that the two procedures were equally effective for increasing extravasation of native albumin, but dMCAO was more effective than craniectomy for increasing extravasation of EBD-bound albumin. Another probable explanation for this difference is that Western blotting, the way by which it was performed in our laboratory, was less sensitive for detecting differences in albumin concentrations than fluorescence-spectrometry was in detecting differences in EBD concentrations. Importantly, these sources of errors have no effect on our conclusion.

In conclusion, we report here that BBB disruption in preclinical stroke models can be an experimental artifact resulting from craniectomy, which is normally required for modeling stroke in preclinical studies. Retrospective analysis of preclinical data may offer additional insight into whether the abrupt exacerbation of ischemia-related BBB disruption caused by craniectomy has led to pseudopositive results regarding the benefits of drugs or therapeutic peptides in preclinical models. Based on our findings, we recommend that craniectomy should be avoided when modeling stroke in future preclinical studies or that the drilling temperature be lowered to minimize the effect of craniectomy on BBB permeability.

## Synthesis

Reviewing Editor: Susanne Wolf, Charite University Hospital Decisions are customarily a result of the Reviewing Editor and the peer reviewers coming together and discussing their recommendations until a consensus is reached. When revisions are invited, a fact-based synthesis statement explaining their decision and outlining what is needed to prepare a revision will be listed below. The following reviewer(s) agreed to reveal their identity: Zhongxiao Fu.

In this manuscript, the authors studied craniectomy induced blood brain barrier (BBB) disruption and cerebral edema in preclinical stroke (dMCAO) models. This work brings our attention that stroke model induced BBB disruption may be due to not only ischemia but also craniectomy surgery. This work is important for preclinical stroke field.

I still have some questions about this work.

1, It will be very helpful to quantify the brain volume of BBB disruption areas in both dMCAO and craniectomy only models. Does craniectomy surgery only induced BBB disruption in superficial layers?

2, Craniectomy surgery induced BBB disruption at 24hr post experiments (Fig2). What about 3hr after surgery?

3, EBD was used to quantify BBB disruption in this paper. Craniectomy induced BBB disruption was not consistent in Fig2A1 and Fig3A, 1500% of control in Fig2A1 and 200% of control in Fig3A. Why there is such a huge variation?

4, Why there are two “craniectomy” groups in Fig5A? What’s the difference?

Why albumin is the same in “craniectomy” group and dMCAO group (Fig5B), although EBD signal is higher in dMCAO group compared to “craniectormy” group (Fig3A) ?

It will also be very helpful to show albumin distribution in the brain in both “craniectomy” group and dMCAO group.

5, In Fig6, authors should mark it clearly which groups were used for comparison. “n.s.” and “**” are between which groups?
